# The effect of time regime in noise exposure on the auditory system and behavioural stress in the zebrafish

**DOI:** 10.1038/s41598-022-19573-y

**Published:** 2022-09-12

**Authors:** Man Ieng Wong, Ieng Hou Lau, Flora Gordillo-Martinez, Raquel O. Vasconcelos

**Affiliations:** Institute of Science and Environment, University of Saint Joseph, Macau, S.A.R. China

**Keywords:** Ecology, Neuroscience, Physiology, Zoology

## Abstract

Anthropogenic noise of variable temporal patterns is increasing in aquatic environments, causing physiological stress and sensory impairment. However, scarce information exists on exposure effects to continuous versus intermittent disturbances, which is critical for noise sustainable management. We tested the effects of different noise regimes on the auditory system and behaviour in the zebrafish (*Danio rerio*). Adult zebrafish were exposed for 24 h to either white noise (150 ± 10 dB re 1 μPa) or silent control. Acoustic playbacks varied in temporal patterns—continuous, fast and slow regular intermittent, and irregular intermittent. Auditory sensitivity was assessed with Auditory Evoked Potential recordings, revealing hearing loss and increased response latency in all noise-treated groups. The highest mean threshold shifts (c. 13 dB) were registered in continuous and fast intermittent treatments, and no differences were found between regular and irregular regimes. Inner ear saccule did not reveal significant hair cell loss but showed a decrease in presynaptic Ribeye b protein especially after continuous exposure. Behavioural assessment using the standardized Novel Tank Diving assay showed that all noise-treated fish spent > 98% time in the bottom within the first minute compared to 82% in control, indicating noise-induced anxiety/stress. We provide first data on how different noise time regimes impact a reference fish model, suggesting that overall acoustic energy is more important than regularity when predicting noise effects.

## Introduction

Anthropogenic noise of variable temporal patterns is increasing in both marine and freshwater ecosystems, being recognized as a global environmental stressor for aquatic wildlife by legislative frameworks worldwide^1^. Noise from traffic, industry, construction work, seismic surveys and recreational activities has become increasingly pervasive, expanding in time and space and affecting many species that rely on sound for orientation and communication^1–4^. These anthropogenic sounds differ greatly from the natural soundscape regarding intensity, spectral composition and temporal features^5–7^. Such altered soundscape may have serious negative effects on organisms including impaired development, heightened physiological stress and behavioural disturbance, thus posing unprecedented risks on animal species, biodiversity and ultimately ecosystems health^2,3,8–11^.

There is a lack of knowledge on how aquatic organisms cope with repeated exposure to noise of different time regimes, i.e. continuous versus intermittent and/or regular versus random. Repeated exposure to an environmental stressor may elicit habituation or sensitization due to augmented or decreased tolerance, respectively^12,13^. Such changes in tolerance are dependent on the intensity, duration and time interval of the stressor^12,14^.

However, only few studies evaluated the effects of noise time regime on fishes^15–18^ and mostly focused on behavioural, developmental and general physiological effects. The behavioural disturbances were more obvious in intermittent treatments, but findings are difficult to compare because of distinct acoustic treatments and developmental stages investigated. For instance, Neo et al.^15^ tested whether sounds with varying temporal structure (continuous versus intermittent at 165 dB re 1 μPa) resulted in different behavioural changes in the European seabass *Dicentrarchus labrax*. All treatments elicited similar behavioural responses, including increased startle responses, swimming speed, group cohesion and bottom diving but intermittent exposure resulted in slower behavioural recovery to pre-exposure levels. Sabet et al.^18^ exposed adult zebrafish *Danio rerio* to a sequence of noise conditions (continuous, fast and slow regular intermittent and irregular intermittent) at circa 122 dB re 1 μPa and found significant increase in startle responses, swimming speed and foraging specially with intermittent treatments. In addition, we tested the effects of different noise time regimes (mimicking shipping activity) on larval zebrafish and suggested that temporal patterns are more important than total exposure duration to down-regulate physiological stress (cardiac rate and yolk consumption)^17^. Although these findings imply that temporal structure of sound is highly relevant in noise impact assessments, more research is needed to identify regimes with less impact on fishes for noise sustainable management strategies^1,11^.

The impact of noise on aquatic life depends on the hearing abilities of organisms. Most anthropogenic noise in the marine and freshwater habitats overlaps with the hearing sensitivity range of fishes and marine mammals, impairing orientation and detection of conspecifics, predators and prey^19,20^. For instance, industrial activities such as pile-driving and shipping generate acoustic energy < 1000 Hz, which overlaps with hearing sensitivity of most fish species^9^. Fish exposed to such acoustic disturbances may suffer inner ear damage, hair cell loss and synaptopathy (including decrease in presynaptic Ribeye b ribbons that enhance neurotransmitter release), resulting on auditory threshold shifts and Noise-Induced Hearing Loss (NIHL)^9,21,22^.

The zebrafish *Danio rerio* (Cyprinidae) is an otophysian species, which possess Weberian ossicles that link the swim bladder to the inner ear enhancing auditory sensitivity especially at higher frequencies^23^. Although zebrafish has become a well-established model in hearing research to investigate inner ear development and ototoxicity^24,25^, there is scarce information on how the acoustic environment can affect its hearing sense^26^. Previously, we reported NIHL and associated hair cell loss with continuous noise exposure in adult zebrafish^27^, but whether specific time regimes in noise exposure result in different auditory effects and the underlying pathological mechanisms remain to be investigated.

The present study relies on a reference model organism, the zebrafish, to evaluate the impact of noise of varying temporal patterns on the auditory system and anxiety-related behaviour. More specifically, we investigated auditory threshold shifts and changes in the inner ear saccule, such as hair cell number and amount of Ribeye b as indicator of synaptopathy^22^. Furthermore, we relied on a standardized assay, the Novel Tank Diving (NTD) test^28,29^, to investigate potential noise-induced anxiety/stress.

## Results

### Noise-induced hearing loss

Adult zebrafish presented an overall auditory sensitivity bandwidth between 100 and 8000 Hz, but only auditory responses within 100–4000 Hz were considered for analysis because they were consistently present in all recordings.

**Figure 1:**
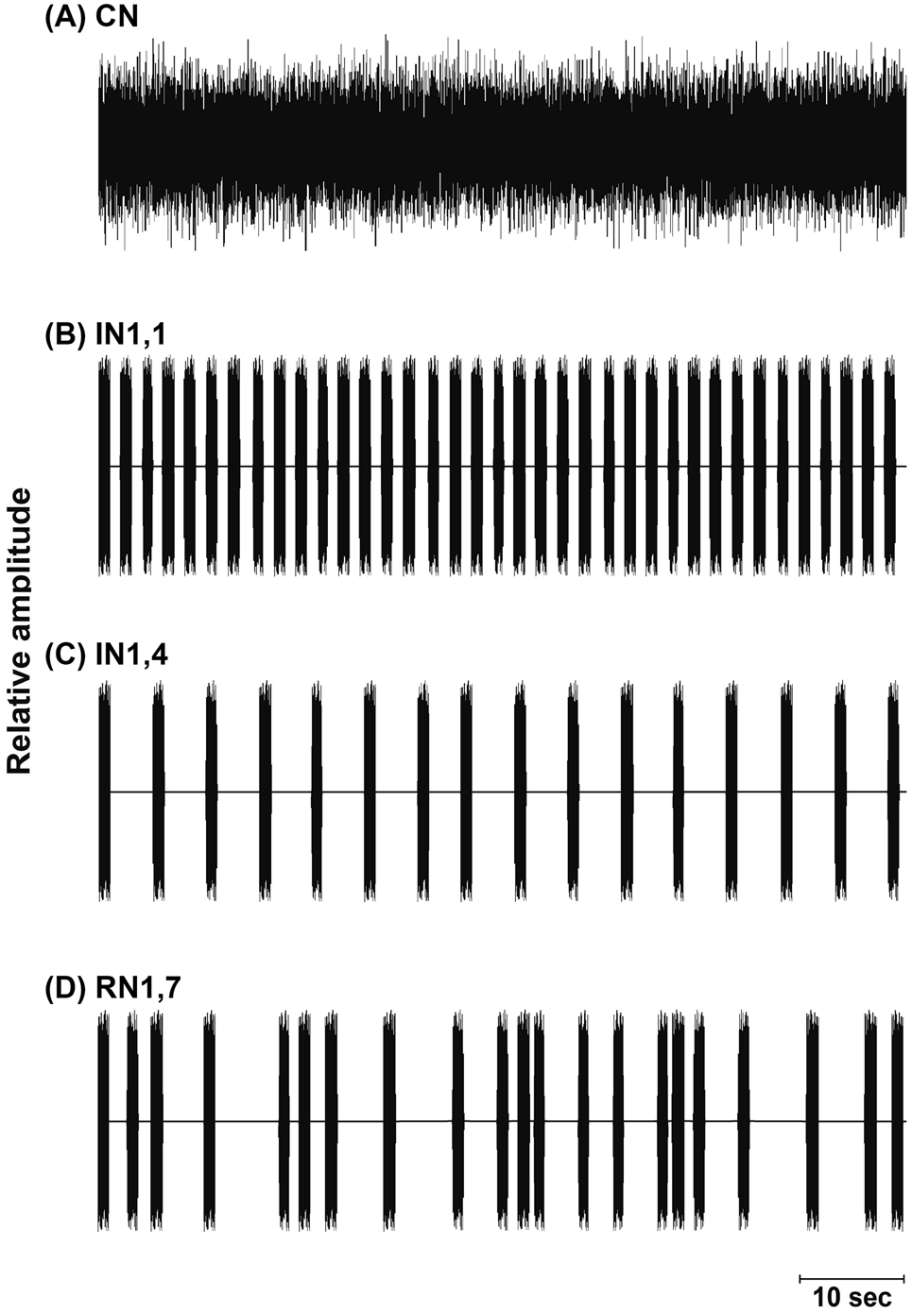
Acoustic treatments used in the experimental design with variable temporal patterns of white noise: (**A**) Continuous noise (CN). (**B**) Intermittent noise with 1 s noise and 1 s silent interval (IN1,1). (**C**) Intermittent noise with 1 s noise and 4 s silent interval (IN1,4). (**D**) Intermittent random noise with 1 s noise and 1–7 s silent (on average 4 s, RN1,7).

**Figure 2:**
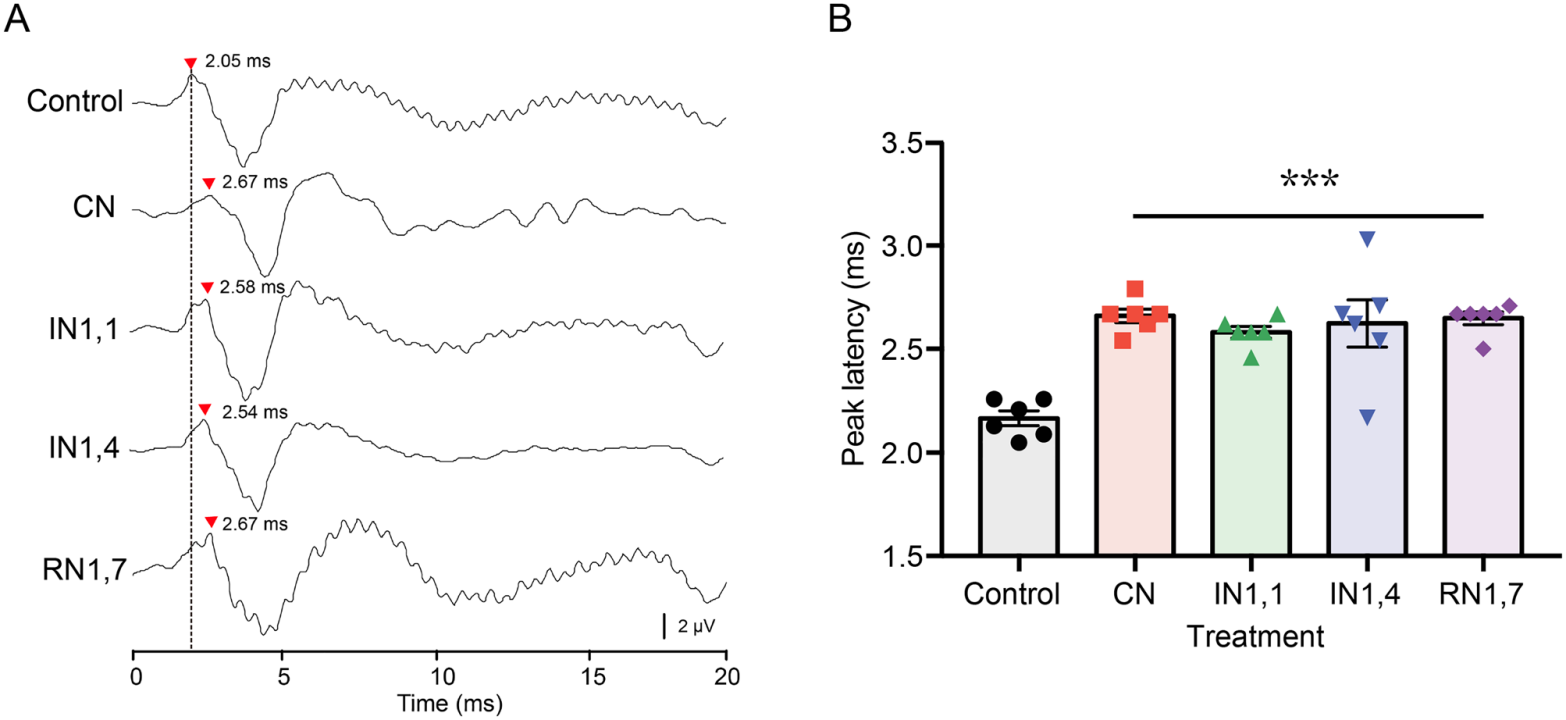
(**A**) Representative Auditory Evoked Potential (AEP) response curves of adult zebrafish under 1000 Hz stimuli (at 120 dB re 1 µPa) after different noise treatments. Arrowheads indicate the peak of each AEP and the corresponding latency with respect to the start of the stimulus. (**B**) Comparison of mean peak latencies ± Standard Error of the Mean (SEM) in response to 1000 Hz stimuli (at 120 dB re 1 µPa) after different noise treatments. Noise treatments caused a delay in response time (F_4,25_ = 12.740, P < 0.0001) with significant differences between control and each noise treatment (***P < 0.001) (CN: continuous noise; IN1,1: intermittent noise with 1 s pulses and 1 s silent; IN1,4: intermittent noise with 1 s pulses and 4 s silent; RN1,7: intermitted random noise with 1 s pulses and 1–7 s silent intervals).

In this study four sound treatments were used with varying noise temporal patterns: continuous noise (CN); intermittent regular noise with a fast pulse rate (IN1,1); intermittent regular noise with a slow pulse rate (IN1,4) and intermittent random noise (RN1,7)—see details in Fig. 1 and “Methods”. Exposure to acoustic treatments for 24 h caused a significant delay in auditory responses measured at the maximum peak of the AEP curve (at 1000 Hz, 120 dB re 1 μPa stimulation) for all noise-treated groups (F_4,25_ = 12.740, P < 0.0001)—Fig. 2. The response latency increased from 2.17 ± 0.04 ms in Control (mean ± SEM, Standard Error of the Mean) up to 2.66 ± 0.03 ms in CN-treated fish.

**Figure 3:**
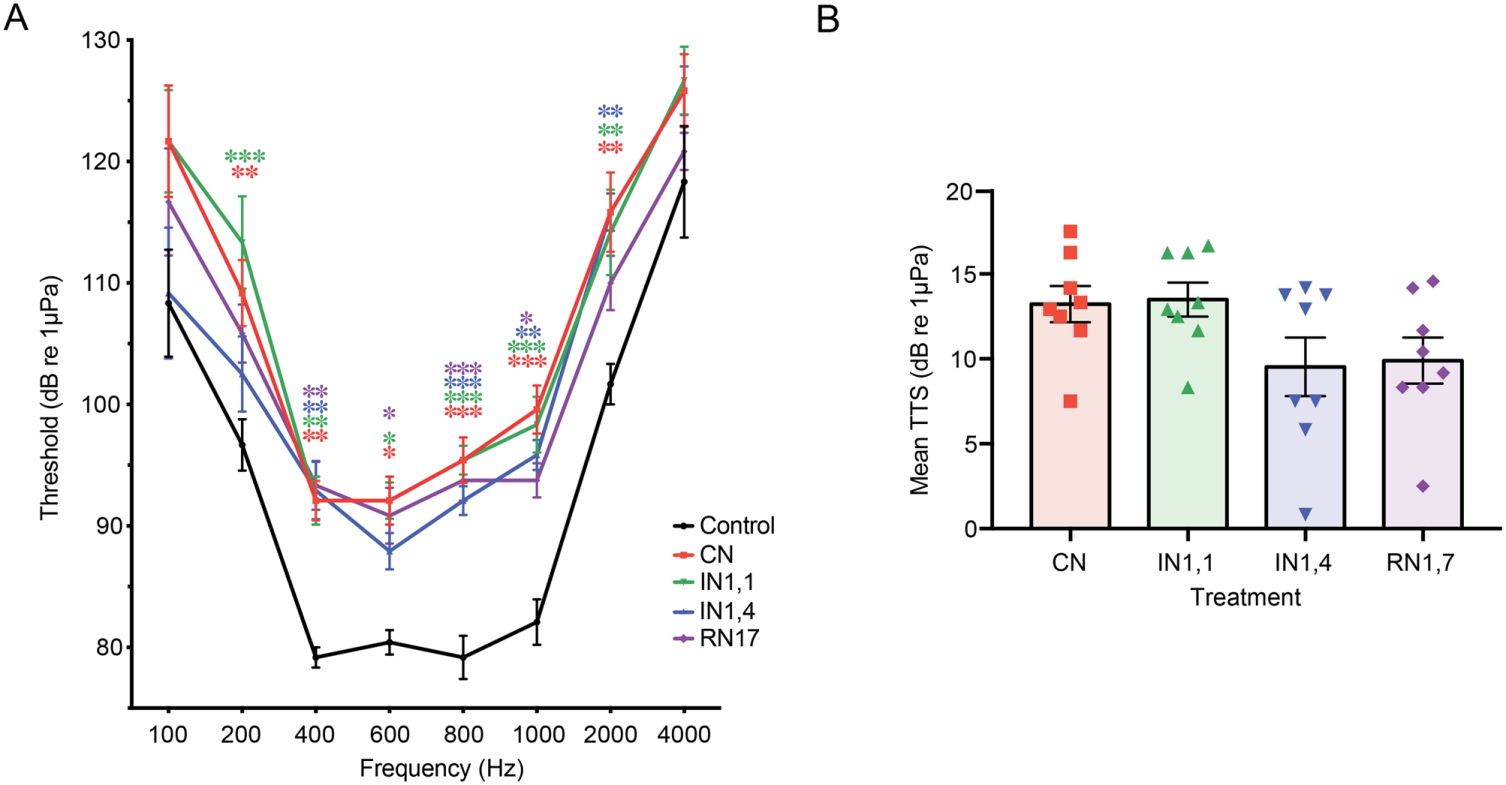
(**A**) Mean auditory thresholds ± Standard Error of the Mean (SEM) of zebrafish after 24 h of either silent lab conditions (control, c. 105 dB re 1 µPa) or white noise of different temporal patterns. Noise induced significant changes in the auditory thresholds (F_4,25_ = 24.710, P < 0.0001) and specific differences between groups at each frequency are indicated based on post hoc tests (*P < 0.05, **P < 0.01, ***P < 0.001). (**B**) Comparison of overall mean Temporary Threshold Shifts (TTS, averaged across frequencies) ± SEM after different noise treatments. Noise exposure induced TTS in all groups but no significant differences were found between them (F_3,28_ = 2.560, P > 0.05). (CN: continuous noise; IN1,1: intermittent noise with 1 s pulses and 1 s silent; IN1,4: intermittent noise with 1 s pulses and 4 s silent; RN1,7: intermitted random noise with 1 s pulses and 1–7 s silent intervals).

Noise treatments caused changes in the auditory thresholds depending on the time regime (F_4,25_ = 24.710, P < 0.0001)—Fig. 3A. Both CN and IN1,1 induced significant increase in thresholds at all frequencies except 100 and 4000 Hz (post hoc tests P = 0.02–0.0001). Individuals exposed to IN1,4 showed increased thresholds within 400–2000 Hz (P = 0.01–0.0032), while the RN1,7 group only presented threshold changes at 400, 600, 800 and 1000 Hz (P = 0.04–0.0014). Overall, the mean Temporary Threshold Shifts (TTS), averaged across frequencies, consisted of 13.2 ± 1.1 (CN), 13.5 ± 1.0 (IN1,1), 9.5 ± 1.7 (IN1,4) and 9.9 ± 1.4 dB re 1 μPa (RN1,7)—Fig. 3B and no differences in TTS were found between noise-treated groups (F_3,28_ = 2.560, P > 0.05).

### Noise-induced effects on the saccular sensory epithelia

**Figure 4:**
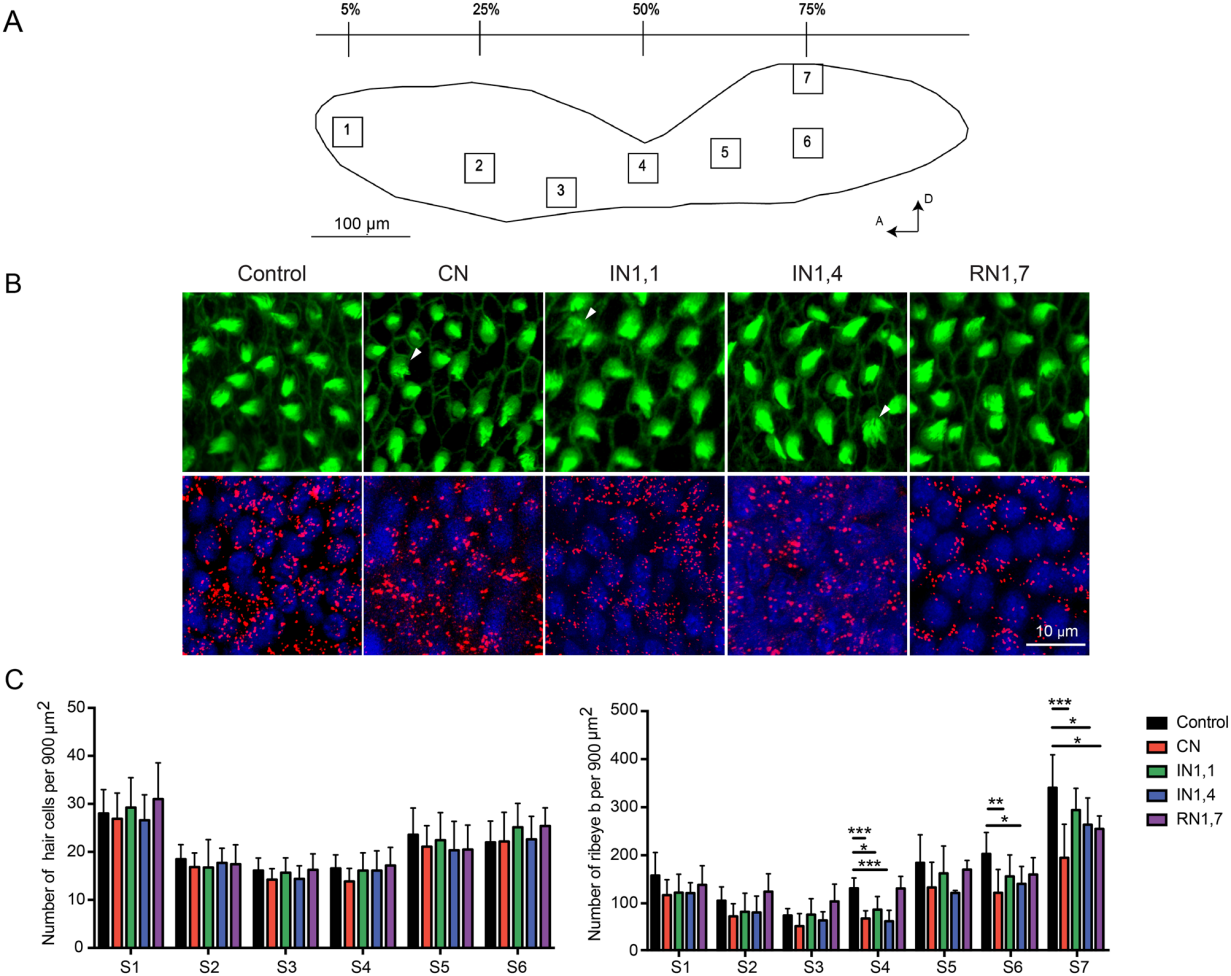
(**A**) Schematic representation of the inner ear saccular epithelium from a zebrafish adult showing the different regions defined for analysis along the rostral-caudal axis. (A: anterior; D: dorsal). (**B**) Representative images showing Phalloidin-stained hair cells (green) and presynaptic Ribeye b puncta (red) from zebrafish saccular epithelia after different noise treatments; white arrow heads indicate disordered hair bundles with splayed stereocilia; cell nuclei (DAPI staining) are also shown in the bottom images. (**C**) Mean ± SEM hair cell number (left) and Ribeye b puncta (right) along the saccular epithelia after different noise treatments (left: F_4,239_ = 2.09, P = 0.083; right: F_4,239_ = 19.80, P < 0.0001). Post hoc differences: *P < 0.05; **P < 0.01; ***P < 0.001. (CN: continuous noise; IN1,1: intermittent noise with 1 s pulses and 1 s silent; IN1,4: intermittent noise with 1 s pulses and 4 s silent; RN1,7: intermitted random noise with 1 s pulses and 1 to 7 s silent intervals).

Detailed morphological analysis of the inner ear saccular epithelia following acoustic treatments revealed noise-induced tissue damage such as splayed hair cell stereocilia and lesions—Fig. 4. However, the objective quantification of such occurrences was difficult, and the structural damage was assessed based on hair cell loss.

Even though the number of hair cell bundles showed a slight reduction among noise-exposed groups (especially in CN and IN1,1 treatments), no statistical differences were found comparing the different experimental groups (F_4,239_ = 2.09, P = 0.083)—Fig. 4.

In order to investigate whether auditory impairment was associated to changes at the synaptic level, we quantified the amount of Ribeye b as an indicator of presynaptic activity. Immunostaining for this protein showed rounded puncta that varied significantly depending on the noise regime considered (F_4,239_ = 19.80, P < 0.0001)—Fig. 4. The amount of Ribeye b was significantly lower in specimens treated with CN compared to control in areas S4, S6 and S7 (P = 0.0014–0.0001), whereas the intermittent treatments also caused a reduction but of lower magnitude in the same regions (P = 0.041–0.0005). The remaining saccular epithelia areas (S1–S3 and S5) showed a decreasing tendency with noise exposure but without significant differences.

### Noise effect on anxiety-related behaviour

We further assessed potential noise-induced anxiety/stress based on the Novel Tank Diving assay. This test relies on the natural tendency of zebrafish to initially dive to the bottom in a novel experimental environment, and the amount of ‘bottom dwelling’ can be interpreted as a measure of anxiety^28,29^.

**Figure 5:**
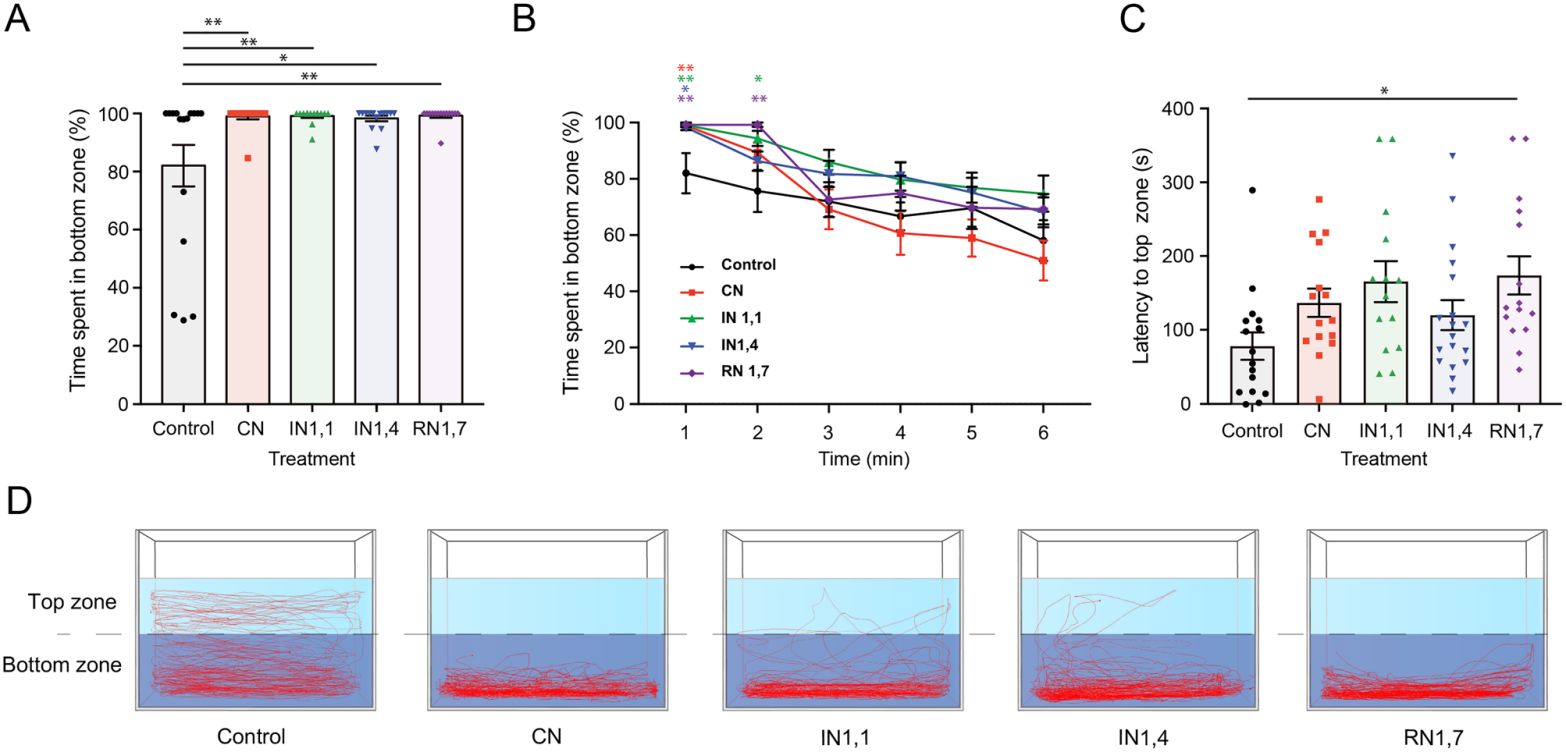
(**A**) Comparison of time spent in the bottom zone (Novel Tank Diving test) by zebrafish adults during the first minute between noise treatment groups (F_4,73_ = 4.930, P = 0.0014). (**B**) Changes in total time spent in the bottom zone (F_4,73_ = 2.771, P = 0.033) and across several time points (F_3.3,238_ = 38.89, P < 0.0001). (**C**) Comparison of movement latency reaching the top zone of specimens previously exposed to different acoustic treatments (F_4,73_ = 2.947, P = 0.026). (**D**) Diagrams showing merged tracking of the first minute of all tested individual fish in the NTD experimental tank (CN: continuous noise; IN1,1: intermittent noise with 1 s pulses and 1 s silent; IN1,4: intermittent noise with 1 s pulses and 4 s silent; RN1,7: intermitted random noise with 1 s pulses and 1–7 s silent intervals).

Our results showed a significant variation in bottom dwelling over time (F_3.3,238_ = 38.89, P < 0.0001), as well as an overall impact of noise treatment (F_4,73_ = 2.771, P = 0.033)—Fig. 5. In particular, noise-treated zebrafish spent significantly more time in the bottom zone (%) during the first minute compared to control (C: 82.04 ± 7.16; CN: 98.98 ± 1.02; IN1,1: 99.1 ± 0.67; IN1,4: 98.28 ± 0.95; RN1,7: 99.27 ± 0.73; F_4,73_ = 4.930, P = 0.0014)—Fig. 5A,D. During the second minute of observation, noise-treated fish were still showing distinct bottom dwelling (F_4,73_ = 4.292, P = 0.0036), but in the following minutes there were no longer differences between groups (minute 3–6: F_4,73_ = 1.186–2.179, P > 0.05)—Fig. 5B.

Moreover, the swimming latency to the top zone of the observation tank was also affected by the noise time regime (F_4,73_ = 2.947, P = 0.026). RN1,7 induced significantly longer latencies compared to control (P = 0.027, overall increment up to 100%), while CN, IN1,1 and IN1,4 showed a latency increment but without significant differences compared to control—Fig. 5C.

Finally, the overall mean velocity (F_4,73_ = 1.969, P > 0.05) and the number of entries in the top zone (F_4,73_ = 1.643, P > 0.05) recorded over the 6 min observation did not change between experimental groups.

## Discussion

This study represents the first attempt to evaluate Noise-Induced Hearing Loss (or NIHL) associated to different temporal patterns in noise exposure in a fish species, the zebrafish, a reference model in hearing research and ecotoxicology. Our findings suggest that the overall acoustic energy is more important than regularity for assessing the negative effects of noise on the auditory system. Moreover, we show that exposure to increased noise levels may cause anxiety-related behaviour in zebrafish based on the Novel Tank Diving assay, suggesting that this standardized should be considered for assessing noise-induced anxiety/stress in fish.

### Effects of noise temporal patterns on hearing loss

In the present study the best hearing range of zebrafish was observed within 400–1000 Hz with mean thresholds around 79–82 dB re 1 μPa. These thresholds were lower than previously reported audiograms for other AB lines^30,31^ but similar to a study conducted with the same species and under the same lab conditions^27^.

The current work evaluates for the first time the impact of noise temporal patterns on the hearing function in a fish species. The results showed that continuous and fast intermittent noise treatments caused the highest impact on auditory sensitivity with threshold shifts of about 13 dB, while slow intermittent and random treatments caused comparatively smaller shifts of circa 10 dB. Noise exposure also caused delayed auditory responses in all treatments with latencies up to 0.5 ms. Altogether, this suggests that the amount of acoustic energy (duty cycle) or overall noise exposure is critical to assess NIHL. The lack or small differences between slow intermittent and random noise treatments suggests that noise regularity is not as critical as overall exposure duration for hearing loss in this model species.

Noise exposure is known to affect the auditory system of fishes causing hearing loss based on a limited amount of studies^27,32–36^. These authors reported significant sensitivity losses within the species best hearing range following exposure to continuous white noise at amplitudes between 140 and 170 dB re 1 μPa for 24 or 48 h (goldfish *Carrasius auratus*^33–36^; catfish *Pimelodus pictus*^34^; fathead minnow *Pimephales promelas*^32^). Recently, we also exposed zebrafish for 24 h to continuous white noise at various amplitudes of 130, 140 and 150 dB re 1 μPa, and reported noise level-dependent temporary threshold shifts of 7, 13 and 22 dB and increased response latencies of 0.4, 0.7 and 1.0 ms (for 130, 140 and 150 dB, respectively)^27^. The results in the present study showed auditory thresholds shifts up to 13 dB and 0.5 ms response latency after continuous noise treatment (under 150 dB) in the same species. Such differences in sensitivity loss were most likely caused by the distinct acoustic treatment conditions. In the prior study by Breitzler et al.^27^, specimens were restrained in a small net box (15 × 15 × 15 cm) to decrease variability in the acoustic field (± 8 dB) and the frequency range of noise playback was 100–1500 Hz. In the present work, zebrafish were freely swimming in a tank with noise level variation of ± 10 dB and a playback bandwidth of 100–3000 Hz. Enclosed spaces such as glass tanks have complex acoustic features due to resonant frequencies and reverberations^37^, so comparisons are always difficult between different setups.

The noise treatments considered in this study consisted of short 1-s pulses interspersed with varying silent intervals similar to impulsive sounds generated in construction activities such as pile-driving^18,38^. This type of acoustic disturbance is substantially increasing in aquatic environments due to exploitation of renewable energy with implementation of wind turbines and construction activities that require bottom-mounted structures^2^. Impulsive sounds are known to impact hearing of marine mammals^38,39^. In fish, Popper et al.^40^ evaluated the effect of impulsive sounds (seismic air guns) in freshwater fishes and showed temporary threshold shifts in some species (northern pike *Esox lucius*, and lake chub *Couesius plumbeus*) but not in others (broad whitefish *Coregonus nasus*), and recovery of affected species occurred within 24 h^40^ with no apparent damage to inner ear hair cells^41^.

Studies that evaluated the effects of noise with varying temporal patterns (or continuous versus impulsive sounds) on the auditory function have focused mostly on mammal species, such as guinea pigs, rats and humans^42–46^. Experimental evidence and legislative standards for occupational noise management suggest that a dose of an impulsive noise is more injurious than the equivalent dose of continuous noise, and that duration and repetition rate are important parameters that affect temporal threshold shifts^42,43^.

We provide baseline information on how noise time regime affects zebrafish hearing based on 24 h noise exposure. Future work should consider evaluating the impact of different noise treatment durations (including shorter periods similar to specific field activities), since this might be critical to predict hearing loss^39^. Besides, complementary research should include analysis of recovering period of auditory function.

### Noise-induced changes in the inner ear

This study investigated whether hearing loss could be explained based on changes in the number of auditory receptors and synaptopathy in the inner ear saccule. The results showed the hair cell number did not decrease significantly with the noise treatments, but a decrease in presynaptic Ribeye b protein was verified especially after continuous exposure.

The effects of noise exposure on the fish inner ear saccule, which plays a major role in hearing in this taxon^47^, have been examined in a few studies. For instance, Smith et al.^48^ exposed goldfish *Carassius auratus* to white noise (170 dB re 1 μPa for 48 h) and identified damage in the central and caudal regions of the saccular epithelia. Schuck and Smith^49^ also used zebrafish to evaluate the effects of noise (pure tone of 100 Hz at 179 dB re 1 μPa for 36 h) and identified hair cell loss and damage in the caudal region of the saccule. However, Casper et al.^50^ exposed hybrid striped bass (*Morone* spp.) and Mozambique tilapia (*Oreochromis mossambicus*) to impulsive pile driving sound at 216, 213, or 210 dB re 1 μPa 2 s, and found hair cell damage only at the highest sound level, suggesting that such acoustic disturbances may have a more significant effect on the swim bladders and surrounding organs rather than on the inner ears of fishes. More recently, Breitzler et al.^27^, exposed zebrafish to 24 h of white noise at 150 dB re 1 μPa and observed maximum of 27% hair cell loss in the anterior saccular epithelia. However, all these studies used either higher sound exposure levels^48–50^ or different acoustic treatment setups^27^, which may explain the contrasting results with our study in terms of noise impact on the inner ear receptors.

The present work revealed a significant reduction in the amount of Ribeye b in specimens treated with continuous noise in the middle and posterior saccular epithelia. Ribeye b expression is an indicator of presynaptic function^51–53^ that has been associated to noise-induced synaptopathy in hair cells^22^. A reduction in Ribeye b protein may lead to presynaptic dysfunction as neurotransmitters are not released so efficiently, thus causing less stimulation of afferent neurons. This phenomenon of synaptopathy is known to be related to decreased auditory sensitivity^22,51–53^. In mammals, cochlear hair cells exposed to moderate noise levels showed reduced presynaptic ribbons^54^. In zebrafish, Wang et al.^31^ identified changes in presynaptic activity in aging zebrafish, showing that age-related hearing loss is related to the change in both hair cell number and ribeye b protein expression. Uribe et al.^55^ exposed larval zebrafish to acoustic trauma and identified lower number of ribbons per hair cell in the neuromasts of noise-treated specimens.

The present work provides first insights on how noise of variable temporal patterns impacts the structure and synaptic function of the inner ear saccule in zebrafish. To complement the current findings, ongoing work focuses on a post-synaptic marker (MAGUK protein) to compare in further detail synaptopathy between groups.

### Effect of noise temporal patterns on behavioural stress

In the present work, we assessed the impact of noise on fish behaviour using a well-established standardized assay for zebrafish, the Novel Tank Diving (NTD)^28,29^. New environments are known to be anxiogenic at the beginning for many animals, including zebrafish^28,29,56^. In this model species, the NTD assay for solitary fish has been used extensively to evaluate anxiety and the effect of anxiolytics^57,58^. This assay exploits the innate tendency of zebrafish to dive to the bottom of a novel environment, gradually exploring the top zone of the tank as they habituate^28,29^.

Our results showed that all noise-treated zebrafish spent more time in the bottom zone during the first minute of observation (> 98% time) compared to control (82%), and a gradual increase in vertical activity was recorded over time. Furthermore, the latency to the top zone was also higher in noise treated groups, especially in intermittent treatments such as RN1,7 that caused a 100% increment. Altogether, these results suggest that noise exposure caused anxiety behaviour, similarly to other anxiogenic treatments. For example, previous work has shown that zebrafish under acute caffeine treatment (100 mg/L, 15 min pre-exposure time) revealed about 57% increase in latency to top zone compared to control^56^.

We adopted a 2-zone “top/bottom” variation of the novel tank as suggested by Egan et al.^59^, and our observations showed that this approach is sensitive enough to identify differences between control and noise-treated groups. However, no differences were found between the various acoustic treatments, suggesting that either the behavioural stress is identical independently of the noise temporal features or the assay is not sensitive enough to distinguish potential differences in acoustic stress. The fact that significant differences were only found within the first 2 minutes of observation indicates that animals were not highly stressed and rapidly habituated to the novel environment. Nevertheless, it would be important to quantify cortisol level in future work to confirm the potential noise-induced physiological stress.

Only a few studies evaluated the effects of noise temporal patterns (continuous versus intermittent) on fish behaviour^15–18^. These studies reported similar behavioural changes in all noise-treated groups, including increased startle responses, swimming speed, group cohesion and bottom diving (European seabass *D. labrax*^15^), but also more significant increase in behavioural patterns (startle responses, swimming speed and foraging) with intermittent treatments (zebrafish^18^). Our results do not show significant differences between continuous and intermittent noise treatments, suggesting that the behavioural stress was identical independently of the noise time patterns similar to Neo et al.^15^. Even though Sabet et al.^18^ used the same species and identical noise temporal patterns to the present study, their experimental design consisted of a sequence of different noise treatments applied to the same individual, making it difficult to compare.

Our findings highlight the importance of considering noise temporal patterns (continuous/fast intermittent versus slow intermittent) when predicting noise effects on the auditory function of fishes. Overall acoustic exposure seems more important than regularity; hence continuous exposure regimes should be avoided as a potential mitigation measure. The vulnerability to acoustic trauma is probably species-specific, but further research using model species such as zebrafish have the potential to unravel fundamental coping mechanisms to acoustic stress in this highly diversified vertebrate group. Finally, the use of a standardized behavioural assay, such as the Novel Tank Diving test, has potential to become a high-throughput tool to assess acoustic disturbance and drug treatments effects, but this requires further validation.

## Methods

### Test animals and husbandry

Wild type adult zebrafish (AB line) were initially obtained from China Zebrafish Resource Center (CZRC, China) and reared at the zebrafish facility of the University of Saint Joseph, Macao. Fish were maintained in 10 L tanks in a standalone housing system (model AAB-074-AA-A, Yakos 65, Taiwan) with filtered and aerated water (pH balanced 7–8; 400–550 μS conductivity) at 28 ± 1 °C and under a 12:12 light: dark cycle. Animals were fed twice daily with live artemia and dry powder food (Zeigler, PA, USA). The fish used in this study were 6–8 months old, both males and females (1:1), with a total length of 2.2–3.1 cm. The total number of specimens tested was 30 for the auditory sensitivity measurements and inner ear morphological analysis (6 fish per experimental group), and 78 for the Novel Tank Diving assay (15-18 fish per group).

All experimental procedures complied with the ethical guidelines regarding animal research and welfare enforced at the Institute of Science and Environment, University of Saint Joseph, and approved by the Division of Animal Control and Inspection of the Civic and Municipal Affairs Bureau of Macao (IACM), license AL017/DICV/SIS/2016. This study was conducted in compliance with the ARRIVE guidelines^60^.

### Noise treatments

Prior to acoustic treatments, all subjects were transferred to 4 L isolation glass tanks that were placed in a quiet lab environment (Sound Pressure Level, SPL: ranging between 103 and 108 dB re 1 μPa) for a minimum of 7 days. These tanks had no filtering system but were subject to frequent water changes, and the light, temperature and water quality were kept similar to the stock conditions. This adaptation period was important to reduce potential effects of noise conditions from the zebrafish housing system.

After this period, groups of six zebrafish were transferred into separate acoustic treatment glass tanks (dimensions: 59 cm length × 29 cm width × 47 cm height; 70 L)—Fig. 1 Supplementary, where they remained 24 h in acclimation. Each tank was equipped with an underwater speaker (UW30, Electro-Voice, MN, USA) housed between two styrofoam boards (dimensions: 3 cm thick × 29 cm width × 47 cm height) with a hole in the centre, positioned vertically in one side of the tank. Another similar sized board was positioned in the opposite side of the tank and fine sand was placed in the bottom to minimize transmission of playback vibrations into the tank walls. Each treatment tank was mounted on top of styrofoam boards placed over two granite plates spaced by rubber pads to reduce non-controlled vibrations.

Four acoustic treatment tanks were prepared for this study to be used alternately between trials and cleaning procedures, but only two were used simultaneously. When two tanks were being used, one contained specimens under acclimation and the other fish under a specific acoustic treatment. The tanks were housed in a custom-made rack and placed at least 1 m apart to minimize acoustic interferences. The tanks were used randomly for the different treatments across the various trials.

The speakers were connected to audio amplifiers (ST-50, Ai Shang Ke, China) that were connected to laptops running Adobe Audition 3.0 for windows (Adobe Systems Inc., USA). After the acclimation period, specimens were exposed to white noise playbacks (bandwidth: 100–3000 Hz) at 150 dB re 1 µPa for 24 h, starting in the morning between 10 and 11 a.m. The bandwidth adopted covered the best hearing range of zebrafish^27^, as well as the frequency range of most anthropogenic noise sources, such as pile driving and vessels^2^.

Sound recordings and SPL measurements were made with a hydrophone (Brüel & Kjær type 8104, Naerum, Denmark; frequency range: 0.1 Hz–120 kHz, sensitivity of − 205 dB re 1 V/μPa) connected to a hand-held sound level meter (Brüel & Kjær type 2270). Noise level was adjusted with the speaker amplifier so that the intended amplitude (LZS, RMS sound level obtained with slow time and linear frequency weightings: 6.3 Hz–20 kHz) was achieved at the centre of the tanks before each treatment. A variation in SPL of ±10 dB was registered in the closest and farthest points (in relation to the speaker). The sound spectra of the noise treatments were relatively flat similar to the setup described in a prior study by Breitzler et al.^27^.

Moreover, the acoustic treatments were calibrated with a tri-axial accelerometer (M20-040, frequency range 1**–**3 kHz, GeoSpectrum Technologies, NS, Canada) with the acoustic centre placed in the middle of the tank. The sound playback generated was about 120 dB re 1 m/s^2^, with most energy in the horizontal axis perpendicular to the speaker, which was verified based on previously described methods using a MATLAB script paPAM^16^.

In this study four sound treatments were used with varying temporal patterns similar to Sabet et al.^18^—Fig. 1: continuous noise (CN); intermittent regular noise with a fast pulse rate—1 s pulses interspersed with 1 s silence (IN1,1); intermittent regular noise with a slow pulse rate—1 s pulses interspersed with 4 s silence (IN1,4) and intermittent random noise—1 s pulses interspersed with 1, 2, 3, 4, 5, 6 or 7 s silent intervals in randomized sequence (RN1,7) leading to a mean interval of 4 s. All intermittent patterns had 5 ms ramps to fade in and fade out pulses for smooth transitions. In the “control” treatment tank, the amplifier connected to the speaker was switched on but without playback.

After each treatment, two specimens were tested for audiometry, two were tested with the NTD assay and another two were euthanized and dissected for inner ear morphological analysis.

### Auditory sensitivity measurements

Auditory Evoked Potential (AEP) recordings were conducted immediately after noise treatments. The AEP recording technique adopted followed previously described procedures^27^. The recordings were conducted in a rectangular plastic tank (50 cm length × 35 cm width × 23 cm height) equipped with an underwater speaker (UW30) positioned in the bottom and surrounded by fine sand. A custom-built sound stimulation system with enhanced performance at lower frequencies (< 200 Hz) was mounted in the centre of the tank wall facing the front and consisted of a vibrating plexiglass disc driven by a mini-shaker (Brüel & Kjær 4810)—see further details in Breitzler et al.^27^. The experimental tank was placed on top of an anti-vibration air table (Vibraplane, KS kinetic systems, MA, USA), which was housed in a walk-in soundproof chamber (2.13 × 2.13 × 2.0 m, 120a-3, IAC Acoustics, North Aurora, IL, USA) constructed as a Faraday cage.

Test adult zebrafish were slightly anaesthetized in 0.12 g/L tricaine methanesulfonate bath (MS-222, Arcos Organics, NJ, USA) buffered with equal concentration of sodium bicarbonate^27,31^. Specimens were positioned in a custom-designed sponge holder, where they remained immobilized with a fine net covering the upper body maintaining normal breathing.

The holder was positioned so that the fish head was just beneath the water surface. Two stainless steel electrodes (0.40 mm diameter, 13 mm length, Rochester Electro-Medical, Inc., FL, USA) were used. The recording electrode was positioned firmly against the skin over the brainstem region, and the reference electrode was placed on the side of the body.

Both sound stimuli presentation and AEP recordings were accomplished using the workstation from TDT (Tucker-Davis Technologies, FL, USA). The AEP recording was fed into a low impedance head stage (RA4LI, TDT) connected to a pre-amplifier (RA4PA, TDT, 20× amplification), band-pass filtered (0.1–1 kHz), and digitized (16 bit, ± 4 mV). The output was then sent to a Multi-I/O processor (RZ6, TDT). Sound stimuli and AEP recordings were controlled with SigGen and BioSig TDT software. Stimuli consisted of tone bursts of 100, 200, 400, 600, 800, 1000, 2000, 4000, and 6000 Hz, presented randomly, with 20 ms duration and 2 ms rise/fall time. Tone stimuli ranged from 2 (100 Hz) up to 80 (6000 Hz) complete cycles and were presented at least 1000 times, half at opposite polarities (180° phase shifted). Prior to each experiment, a hydrophone (Brüel & Kjær 8104) connected to a sound level meter (Brüel & Kjær 2270) was used for calibrating the stimuli at the position that would be occupied by the fish head in the recording tank. Although it would be ideal to calibrate the system also in particle motion, this was not possible due to the size of available accelerometer and setup constraints. Nevertheless, the information provided should be enough for a comparison between experimental groups.

For each frequency, tones were initially presented at 140 dB re 1 µPa and then in 2.5 dB decreasing steps. The auditory threshold was defined as the lowest SPL at which a visible and repeatable AEP response was identified in at least two averaged waveforms. The validation of an auditory response was based on at least two out of three possible criteria: (1) general waveform shape matching response from previous sound level (visual inspection); (2) presence of a delay or increased latency in the auditory response measured in a consistent AEP peak; and (3) presence of a spectral peak in the FFT analysis of the auditory response that is double of the stimulation frequency.

The peak latency of auditory responses was determined for each individual at 1000 Hz and 120 dB re 1 µPa stimulation. This measure was determined based on the time interval between the tone stimulus onset and the maximum peak of the AEP response. An overall mean TTS was also calculated for each noise treatment group based on the mean auditory thresholds obtained for the several frequencies subtracted by the mean thresholds obtained from control group at the same frequencies.

### Inner ear morphological analysis

Saccular epithelia were obtained from selected specimens immediately following noise treatments. Fish were euthanized with overdose MS-222 (300 mg/L) buffered with sodium bicarbonate. We followed the methods previously described to extract zebrafish inner ear sensory epithelia^61^ and double stain saccular hair cells and pre-synaptic Ribeye b^31^.

Firstly, the fish heads were cut and the ventral part of the skull was cracked open, and then fixed in 10% neutral buffered formalin solution (Sigma, USA) at 4 °C overnight. After fixation, samples were thoroughly rinsed with PBS and then dissected under a dissecting stereomicroscope (Stemi 2000CS, Zeiss).

In order to stain the hair cells (HC) and Ribeye b, saccular epithelia were first permeabilized with 1% Triton X-100 RT for 2 h, followed by blocking with 10% goat serum in PBS and incubated with primary antibody against the Ribeye b protein (mouse anti-zebra Ribeye b monoclonal antibody, gift from Dr. T. Nicolson, Stanford University, CA, USA; 1:5000) at 4 °C overnight. In the following day, samples were firstly rinsed with 1% PBST for 2 h, then incubated with Alexa Fluor 647 IgG2a secondary antibody (Invitrogen, USA; 1:500) and Alexa Fluor 488 phalloidin (Invitrogen, USA; 1:500) for 2 h, and finally rinsed in PBS for 30 min. Saccular epithelia were further labelled with DAPI (Invitrogen, USA; 1:1000) for visualization of cell nuclei and whole mounted with Fluoromount-G (Southern Biotech, USB).

The tissue samples were imaged with a confocal imaging system (STELLARIS 5 LIA, Leica Microsystems CMS GmbH) using Leica Application Suite software X (LAS X, Leica Microsystems Leider Lane) and further analysed with image J (Version 1.53e, National Institutes of Health, USA). Both HC bundles and Ribeye b puncta were quantified manually in seven non-overlapping squared regions of 900 mm^2^ located across the length of the rostral-caudal axis of the saccule, as shown in Fig. 4A and according to previous studies^31^. All HC bundles and Ribeye b puncta within or overlapping the square outlines were included in the counts.

### Novel Tank Diving assay

To evaluate the impact of noise treatments on swimming activity and anxiety-like behaviour, two fish from each acoustic treatment tank were tested with the Novel Tank Diving (NTD) assay^62^ immediately following the treatments. NTD was tested in a rectangular-shaped glass transparent tank (26 cm length × 13 cm width × 19.5 cm height) filled with 4 L of system water. To perform NTD test, a single fish was simply transported to the experimental tank and the behaviour recorded with a digital camera (SONY HDR-PJ675, Japan) for 6 min. The water from the experimental tanks was replaced between trials to avoid potential effects from chemical cues.

Videos were analysed using EthoVision XT 15 (Noldus Information Technology, Netherlands) to generate a tracking line for each individual fish. To measure the vertical exploratory activity, the tank was divided into two equally sized areas (top and bottom), as shown in Fig. 5D. Specific parameters were calculated based on the overall 6 min recording: mean velocity (cm/s)—overall swimming distance divided by recording time; latency to the top zone (s)—time interval between the beginning of the trial until the first entry in the top zone; percentage (%) of time spent in the bottom zone—amount of time in the bottom zone per each consecutive 60 s, and the number of top zone entries.

### Data analysis

The effects of noise treatment on auditory thresholds were tested with Repeated Measures Analysis of Variance (RM ANOVA). The noise treatment was considered a between-subject factor, while the different frequencies were the repeated measures (within-subject factor). The impact of noise treatment on the peak latency was tested with one-way ANOVA. The effect of the acoustic treatment on hair cell number and Ribeye b punctua were tested with two-way ANOVA, with treatment and epithelial region as factors.

Regarding the behavioural tests, the effect of noise treatment on the time spent in the bottom zone was tested with RM ANOVA, considering noise treatment as a between subject factor, and different time points of the trial as repeated measures. Furthermore, time spent in the bottom (per min), the mean velocity, latency to the top zone, percentage (%) of time spent in the bottom zone (per 60 s), and the number of entries in the top zone were compared between treatments using one-way ANOVA.

ANOVAs were followed by Tukey’s pairwise comparison post hoc test. All assumptions for parametric analyses were confirmed through the inspection of normal probability plots and by performing the Levene’s test for homogeneity of variances. Graphs and statistical tests were performed using Prism 7 for Mac OS X (GraphPad, USA) and SPSS v26 (IBM Corp. Armonk, USA).

## Supplementary Information

Supplementary Figure 1.

## Data Availability

The datasets generated and/or analysed during the current study are not publicly available due the type of work and analysis carried, but are available from the corresponding author on reasonable request.
