# Supplementary material for: The effect of time regime in noise exposure on the auditory system and behavioural stress in the zebrafish
Source: Sci Rep. 2022 Sep 12;12:15353. doi: 10.1038/s41598-022-19573-y (PMC9468136; doi:10.1038/s41598-022-19573-y)
Supplement: Supplementary file 1 — Supplementary Figure 1. [file 41598_2022_19573_MOESM1_ESM.pdf]

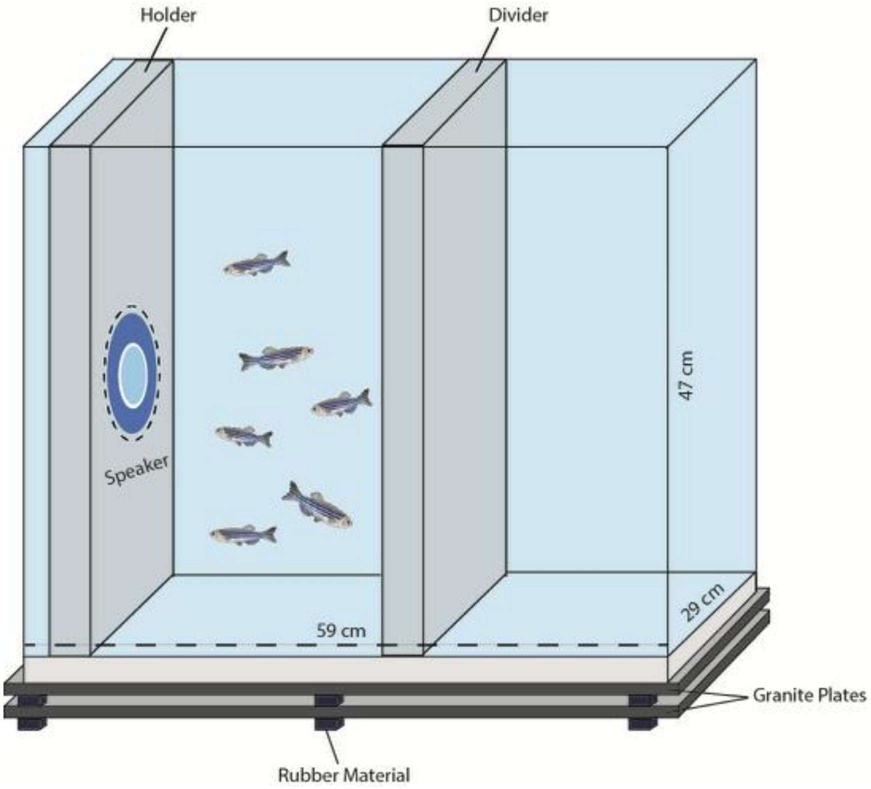

Fig. 1 Supplementary. Experimental playback setup. Tanks used for noise exposure were mounted on top of a Styrofoam plate and two granite plates (1.5 cm thinkness). Granite plates were spaced by rubber materials. The underwater speaker (UW30, Electro-Voice, MN, USA) was mounted vertically using a custom built Styrofoam holder. Another Styrofoam divider was placed opposite to the speaker.
